# Measurement properties of the Flu-Like Symptom Index from the Hepatitis Physical Symptom Severity Diary

**DOI:** 10.1007/s11136-013-0609-0

**Published:** 2013-12-31

**Authors:** Susan Mathias, Ross D. Crosby, Martha S. Bayliss, Gilbert L’Italien, Sandhya Sapra

**Affiliations:** 1 Health Outcomes Solutions, Winter Park, FL USA; 2 Neuropsychiatric Research Institute, Fargo, ND USA; 3 University of North Dakota School of Medicine and Health Sciences, Grand Forks, ND USA; 4 QualityMetric Incorporated, Lincoln, RI USA; 5 Bristol-Myers Squibb, Princeton, NJ USA; 6 Yale University School of Medicine, New Haven, CT USA

**Keywords:** Hepatitis C virus, Flu-Like Symptom Index, Hepatitis Physical Symptom Severity Diary, Validation, Measurement properties

## Abstract

**Purpose:**

Chronic Hepatitis C (CHC) Virus infection is a serious health issue in the US. Standard treatment involves peginterferon alpha and ribavirin, often associated with adverse side effects including flu-like symptoms. These adverse effects are common reasons for the discontinuation of treatment and therefore represent a major obstacle in the effective treatment of CHC.

**Methods:**

The Hepatitis Physical Symptom Severity Diary, a newly developed patient-reported outcome measure for assessing physical symptoms in CHC patients, was recently developed. It contains four questions addressing flu-like symptoms [the Flu-Like Symptom Index (FLSI)]. Measurement properties of the FLSI in CHC patients were assessed using data from two randomized clinical trials.

**Results:**

Exploratory factor analysis using data from baseline and the last visit while on treatment supported a single-factor solution for the FLSI. Internal reliability and test–retest reliability are acceptable (Cronbach’s alpha range 0.73–0.81; intraclass correlation coefficient range 0.85–0.97), and correspondence to several similar constructs was acceptable. The FLSI score was higher among those with investigator-reported flu-like symptoms (mean = 4.1) versus those without (1.4), although not statistically significant (*p* = 0.12). Responsiveness of the FLSI was moderate, as measured by standardized effect sizes and response means, and the minimum important difference (MID) was estimated at 2.5–3.0 points.

**Conclusions:**

While additional research should be conducted to evaluate validity with more closely related constructs and to utilize anchor-based methods for estimating the MID, data suggest that the FLSI has acceptable measurement properties and can be an effective tool in assessing flu-like symptoms in CHC patients.

## Introduction

Chronic Hepatitis C (CHC) virus infection is the most common blood-borne pathogen and affects approximately 3.2 million individuals in the USA [1, 2], and as many as 130–170 million world-wide [3]. Up to 70 % of individuals with CHC develop chronic liver disease, and up to 20 % develop cirrhosis of the liver [1, 3]. CHC is the leading cause of liver transplantation in the USA [4]. Each year between 8,000 and 10,000 individuals die as a result of CHC in the USA [1].

Treatment of CHC typically involves the administration of peginterferon alfa (alfa) and ribavirin (RBV), usually associated with a variety of physical symptoms including fatigue, headache, fever, and rigors [5, 6]. Side effects are often the primary reason that individuals reduce dosage or discontinue treatment altogether [6, 7]. In one study of alfa/RBV, of the more than 1 in 5 who withdrew, 32 % did so because of side effects [8]. The most commonly cited side effects were fatigue (54 %), headaches (47 %), fever (43 %), and joint pain (42 %). In another study comparing different doses of alfa and RBV, 13–14 % of all patients in the study discontinued due to an adverse event. Although the specific adverse events that led to discontinuation were not detailed, in the high- and low-dose groups, some of the most commonly reported physical symptoms included fatigue (62–64 %), fever (44–46 %), headache (58–62 %), rigors (45–48 %), and joint pain (48–56 %) [9]. In a recent study of 150 CHC patients, the majority of treatment-experienced patients (76 %) experienced flu-like symptoms 1–3 days after every injection, and almost half (44 %) of those patients missed at least one day of work because of them [10]. In that same study, the number of “flu-days” was reported by patients as the most important reason for nonadherence after lack of efficacy and depression [10]. In another study using patient charts to investigate reasons for alfa discontinuation, 52 % of patients who withdrew from treatment did so because of adverse events, such as fatigue, muscle aches, and nausea [11. Discontinuation or disruption of treatment in CHC is associated with lower rates of viral suppression, and therefore, consideration of side effects is important when selecting an appropriate treatment for patients [12].

While new direct-acting anti-virals have been approved for the treatment of CHC, these new anti-virals are added to the previous alfa/RBV regimens and hence bring incremental side effects. Until recently, no patient-reported outcomes (PRO) measures have been available to evaluate the presence and severity of physical symptoms associated with CHC treatment. However, within CHC studies, there is a growing understanding regarding the importance of collecting measures beyond the usual clinical outcomes, to include those reported by the patient [13]. A recent review of patient-centered, qualitative research revealed similar findings based on patients’ experience of symptoms and side effects in CHC. Flu-like symptoms, specifically, were among the concepts mentioned spontaneously by the patients studied. This confirms observations by clinicians and experts regarding the impact of CHC and the available treatments [14–19]. In a 2012 review, a gap in the current literature was noted regarding PRO measures for CHC patients. Specifically, the authors called for future research to develop a PRO measure that could be used to assess the most common flu-like symptoms [20].

To this end, a brief patient-reported measure, the Hepatitis Physical Symptom Diary (HPSS-D), which asks patients about the physical symptoms of CHC and its treatment, was developed [21]. While physical symptoms are important during CHC treatment, flu-like symptoms (a constellation of physical symptoms) are especially critical given the impact of these symptoms on treatment initiation and discontinuation. Hence, the decision was made to focus on defining and examining the psychometric properties of the Flu-Like Symptom Index (FLSI), a domain of the HPSS-D that assesses the severity of flu-like symptoms in patients treated for CHC. Within the HPSS-D are 4 items that were found to represent flu-like symptoms based on clinical input and support by factor analysis. The objective of this study is to assess the reliability, validity, and responsiveness of the FLSI in patients with CHC. Additionally, the minimum important difference (MID) was established to aid in its interpretation.

## Methods

As part of the effort to develop the HPSS-D, six focus groups (three from the Mayo Clinic in Rochester, Minnesota and three from Inova Fairfax Hospital in Falls Church, Virginia) with a total of 52 CHC patients were conducted with treatment-naïve patients (*n* = 2 groups, 9 males/5 females, mean age = 53 years, 86 % white), patients currently undergoing treatment (*n* = 2 groups, 8 males/9 females, mean age = 51 years, 76 % white), and patients that previously received treatment (*n* = 2 groups, 16 males/5 females, mean age = 51 years, 81 % white). All six focus groups were conducted by experienced moderators and licensed clinical psychologists. So as not to introduce bias, participants were asked open-ended questions designed to ascertain what symptoms they experience related to CHC and CHC treatment, as well as the impact of CHC on their lives. A semi-structured discussion guide was used that included questions like, “What symptoms of hepatitis C have you experienced?”, “How has your life changed since you developed hepatitis C?”, and “What has your hepatitis C treatment experience been like?” Many participants reported that they had to stay in bed for 2–3 days following treatment due to fatigue and flu-like symptoms. Patients also discussed the psychological or emotional side effects of treatment (depression, sadness, hostility, etc.) as well as the impact that treatment had on their cognitive function (forgetfulness and difficulty in concentrating). Patients indicated that among their physical symptoms, fatigue and flu-like symptoms had the most significant negative impact. From this input as well as feedback from clinicians, an initial version of the HPSS-D was developed. Cognitive debriefing interviews were then conducted on 14 additional patients with CHC. Participants were asked to provide feedback on the draft version of the HPSS-D, and using that feedback, the HPSS-D was finalized. For instance, both a 0–5 scale and a 0–10 scale were tested, and patients preferred the 0–10 scale, stating that it provided them more choices than the 0–5 point scale. The four questions in the HPSS-D that comprise the domain of FLSI represent the first PRO measure to specifically assess flu-like symptoms in patients with CHC.

**Fig. 1:**
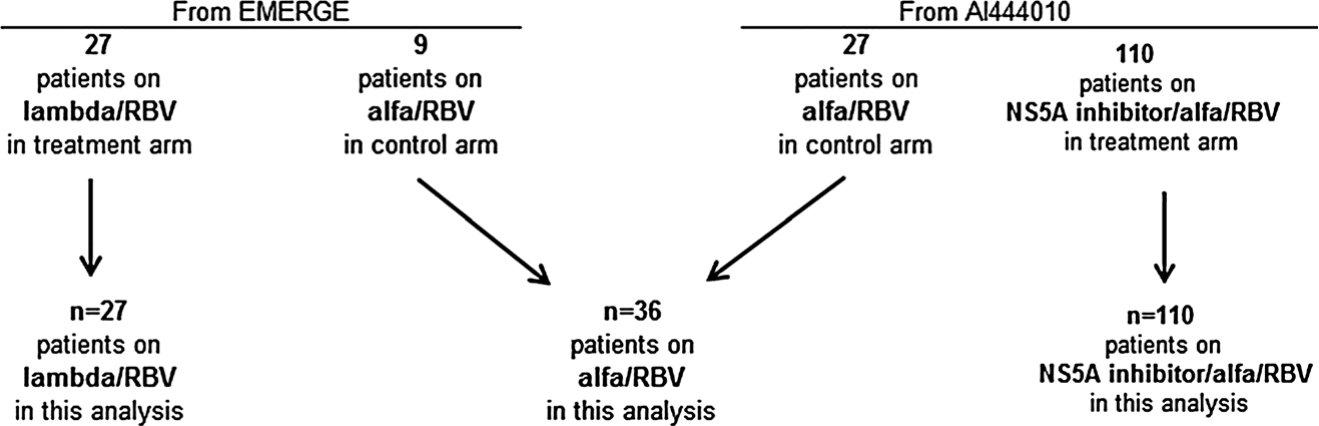
Study design

This study employs data collected on the HPSS-D from CHC patients participating in two separate randomized clinical trials (Fig. 1). At multiple time points, the HPSS-D, which includes the FLSI, was administered. Using this information, as well as clinical data and data available from other concurrent measures, several methods were employed to assess the measurement properties of the FLSI domain.

### Hepatitis Physical Symptom Severity Diary and Flu-Like Symptom Index

The HPSS-D is a 14-item daily diary measure designed to assess the presence and severity of physical symptoms associated with CHC and/or CHC treatment. Each of the 14 items uses a numeric rating scale response option ranging from 0 (no symptom or problem) to 10 (worst symptom or problem imaginable). The diary has a recall period of the past 24 h and is completed daily. Four of the items within the HPSS-D (fever, chills, muscle aches and pain, and joint pain) comprise the FLSI. A score for the FLSI is calculated by averaging responses for these four items. Higher scores indicate worse flu-like symptoms.

### Patients

Patients from two separate Phase 2b randomized clinical trials evaluating two treatments for CHC were asked to complete the HPSS-D. The first Phase 2b trial (EMERGE, NCT#: NCT01001754) included treatment-naive patients with CHC genotype (GT) -1, -2, -3, or -4. A total of 36 patients were included [27 in the treatment group receiving peginterferon lambda-1a (BMS-914143) plus RBV and 9 in the control receiving alfa/RBV]. The HPSS-D was administered for 7 consecutive days at three time points: baseline, week 4 and week 12. (Although patients completed the HPSS-D for seven consecutive days at each time point, for comparability with the second study where it was only collected for one day, data from only day 1 of the HPSS-D were used). Additional measures administered in the EMERGE trial included the Beck Depression Inventory II (BDI-II) [22–24], the Fatigue Severity Scale (FSS) [25, 26], and investigator reports of flu-like symptoms and musculoskeletal symptoms. The BDI-II and FSS were administered at baseline, while reporting of treatment side effects was obtained at each time point. The second study (A1444010, NCT#: NCT01125189) included 137 treatment-naïve patients (110 in the treatment group receiving an NS5A inhibitor (BMS-790052) and 27 in the control arm receiving alfa/RBV) with CHC GT -1 or -4, where the HPSS-D was administered once at baseline, and then again at weeks 4 and 12. Although the entire HPSS-D was administered to patients in both studies, our analysis focused specifically on the FLSI.

### Statistical methods

Response characteristics of individual items were evaluated at baseline and week 12, including floor and ceiling effects. Statistical analyses regarding validity and reliability were conducted on the FLSI using data from baseline, and the last visit while on treatment (most often at week 12) pooled across studies, unless otherwise noted. Exploratory factor analysis (EFA) was conducted on data from baseline as well as from the last visit while on treatment to evaluate the number of underlying constructs in the FLSI. The EFA employed the maximum likelihood extraction method.

Internal consistency reliability is a measure of how well different items correlate with each other to constitute a multi-item scale. Internal consistency was assessed using Cronbach’s alpha coefficient and corrected item-total correlations. Test–retest reliability, or the reproducibility of the FLSI, was evaluated using the intraclass correlation coefficient (ICC) using the 7 daily FLSI scores at baseline from the EMERGE trial. Results were compared with the generally accepted standard of 0.70 for both Cronbach’s alpha and the ICC, and to a threshold of 0.30 for item–total correlations [27].

To demonstrate the convergent validity of the FLSI, its correspondence with similar constructs was evaluated using Pearson correlation coefficients [28]. The similar constructs included the BDI-II and FSS, as well as investigator-reported side effects of treatment. A high level of convergent validity would indicate that the FLSI identifies symptoms to the same degree of these other constructs. Convergent validity was computed using baseline data, last visit while on treatment data, and the change from baseline to the last visit.

Known groups validity was evaluated by comparing FLSI scores between groups based on physician ratings of flu-like symptoms and musculoskeletal symptoms [28]. Physician ratings were used since no gold standard exists for these outcomes.

To gauge the responsiveness of the FLSI to clinical changes, the standardized effect size (SES) and the standardized response mean (SRM) were computed using data from baseline and last visit while on treatment: These measures of the sensitivity to change can be interpreted as small (0.20–0.49), medium (0.50–0.79), and large (≥0.80) [29, 30].

Distribution-based methods were used to compute estimates of the minimum important difference (MID), including the standard error of the mean (SEM) and the 0.50 effect size [31–33]. Cumulative distribution function curves were also created to aid in interpretation of the data. Anchor-based methods [34] for estimating the MID could not be utilized because no appropriate anchors were available.

## Results

### Patient characteristics

The analysis dataset included interim data from 137 subjects from study AI444010 and 36 subjects from the EMERGE study. As Table 1 shows, the sample was 78.0 % white, 19.7 % black or African American, and 0.6 % Asian, with the remainder either of another race or race not reported. Sixty-four percent of the sample was male, and the mean age was 50 ± 10 years (range 18–67 years). A total of 32 patients (18.5 %) reported a history of alcohol abuse/dependence, 55 (31.8 %) reported a history of drug abuse/dependence, and 54 (31.2 %) reported a history of psychiatric problems. HCV genotype for the A1444010 study included 107 (78.1 %) with genotype 1A, 27 (19.7 %) with genotype 1B, and 3 (2.2 %) with genotype 4. HCV genotype for patients from the EMERGE study included 32 (88.9 %) with genotype 1, 2 (5.6 %) with genotype 2, 1 (2.8 %) with genotype 3, and 1 (2.8 %) with genotype 4.

**Table 1:** Demographic characteristics of the study cohort (*N* = 173)

*Age*	
Mean (SE)	50.4 (10.3)
Range	18–67
*Gender (N, %)*	
Male	110 (63.6)
Female	63 (36.4)
*Race (N,* *%)*	
White	135 (78.0)
Black	34 (19.7)
Asian	1 (0.6)
Other/no answer	3 (1.7)

### Response characteristics

At baseline, a large percentage of patients had responses at the lowest end of the response options, suggesting a potential floor effect for vomiting (94 %), fever (87 %), chills (84 %), nausea (83 %) and loss of balance (83 %). At week 12, floor effects for fever (70 %) and vomiting (83 %) were evident. Ceiling effects were almost nonexistent at both baseline and week 12, as no more than 1 % of patients reported the maximum score for any HPSS-D item.

### Exploratory factor analysis

**Fig. 2:**
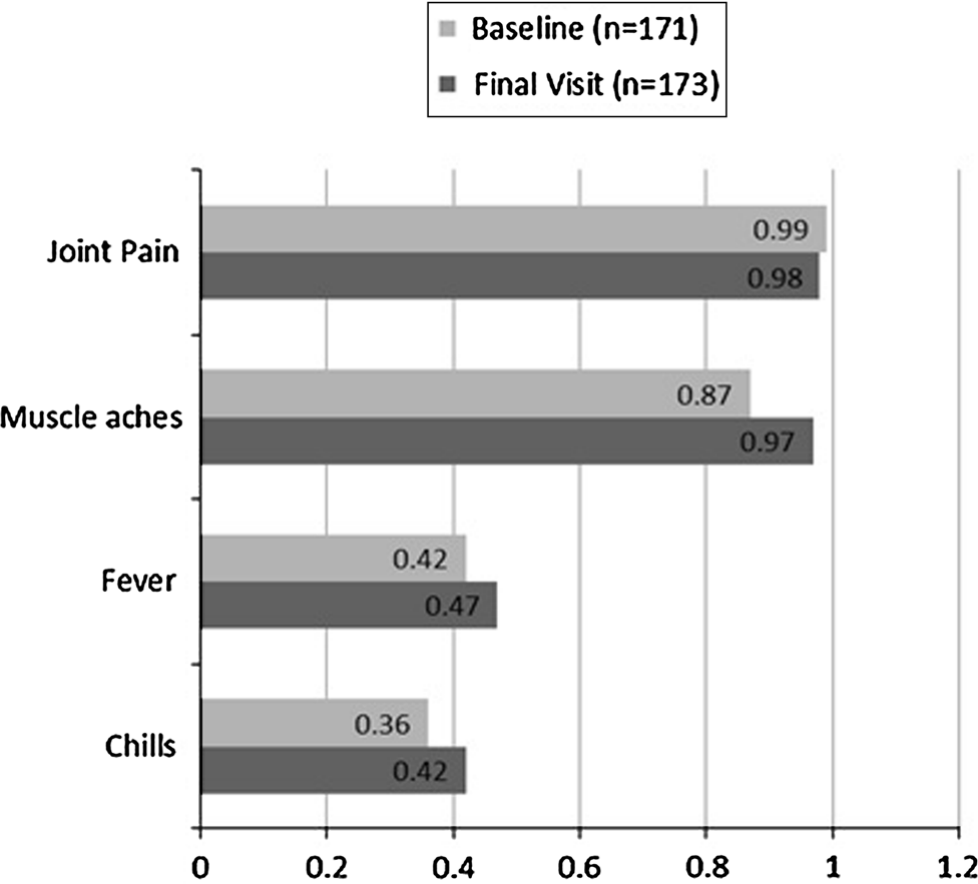
Factor loadings in 1-factor model for the Flu-Like Symptom Index

The initial pool of items to be included in the EFA was based upon clinical relevance to flu-like symptoms. Items were retained or discarded on the basis of factor loadings in an iterative process until a final model was identified. Based on the Kaiser–Guttman rule, the EFA using data from both studies at baseline and the last visit while on treatment supported a single-factor solution for the FLSI (Table 2). At each time point, only the first factor had an eigenvalue greater than 1, and the ratio of the first eigenvalue to the second was close to 3:1. A one-factor solution explained 58 % of the variance at baseline and 66 % of the variance at the last visit while on treatment. The range of factor loadings for the one-factor solution was 0.355–0.999 at baseline and 0.423–0.980 at the last visit while on treatment (Fig. 2). Loadings were lower for chills and fever, but still above or near the acceptable threshold of 0.40.

**Table 2:** Flu-Like Symptom Index exploratory factor analysis at baseline, total variance explained

Factor	Initial eigenvalues			Extraction sums of squares loadings		
	Total	% of variance	Cumulative %	Total	% of variance	Cumulative %
*Baseline*						
1	2.337	58.414	58.414	2.050	51.252	51.252
2	0.841	21.014	79.428			
3	0.695	17.365	96.793			
4	0.128	3.207	100.000			
*Last visit*						
1	2.622	65.553	65.553	2.305	57.621	57.621
2	0.848	21.198	86.751			
3	0.485	12.131	98.882			
4	0.045	1.18	100.000

### Reliability

At baseline, Cronbach’s alpha coefficient for the FLSI was 0.73, above the generally accepted standard of 0.70. All corrected item-to-total correlations were greater than 0.30, indicating that all items are measuring the same construct as other items in the scale. The corrected item-to-total correlations ranged from 0.34 for chills to 0.85 for joint pain. At the last visit while on treatment, results again showed acceptable internal consistency reliability (Cronbach’s alpha = 0.81) and corrected item-to-total correlations ranging from 0.52 (chills) to 0.85 (joint pain). Deletion of any single item at baseline or the last visit while on treatment did not result in an increase in Cronbach’s alpha (data not shown). ICC values were calculated for both studies at those time periods. In every case, the ICC was above the generally accepted standard of 0.70 (0.85–0.97 for EMERGE, 0.82–0.91 for A1444010).

### Convergent validity

**Fig. 3:**
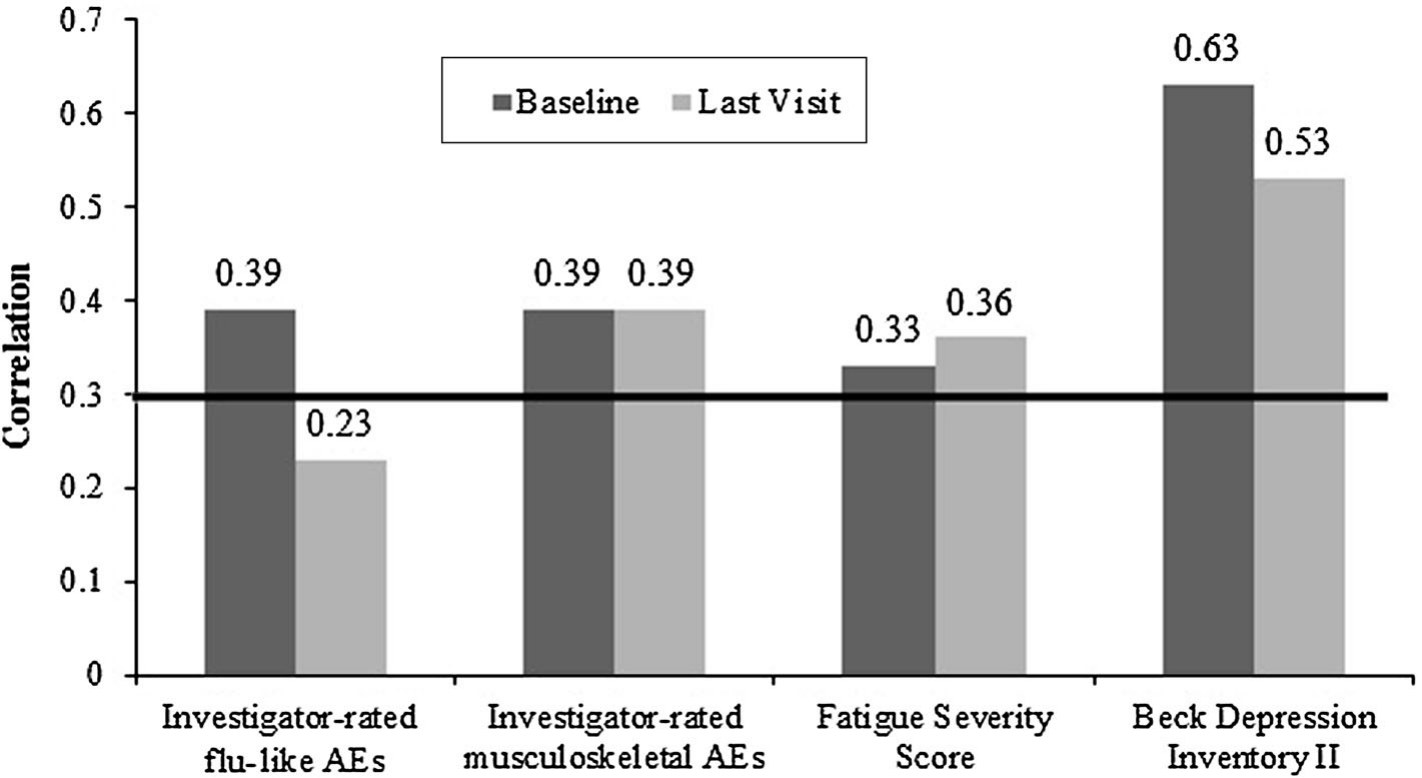
Correlations between the Flu-Like Symptom Index and the investigator-rated symptoms at baseline and the last visit

At baseline, the correlations between the FLSI and the investigator-rated flu-like symptoms and the investigator-rated musculoskeletal symptoms were moderate (*r* = 0.39, *p* = 0.02 for both). The correlation coefficients between the FLSI and the FSS and BDI-II were 0.33 (*p* = 0.06) and 0.63 (*p* < 0.001), respectively. Using data from the last visit while on treatment, results were similar or slightly lower than at baseline, as shown in Fig. 3. Specifically, correlations of 0.23 (investigator-reported flu-like symptoms, *p* = 0.17), 0.39 (investigator-reported musculoskeletal symptoms, *p* = 0.02), 0.36 (FSS, *p* = 0.04), and 0.53 (BDI-II, *p* = 0.001) were found with the FLSI at the last visit. The FLSI was found to have low and nonsignificant correlations with physician-rated anemia (*r* = −0.095, *p* = 0.58) and moderate correlations with the reported flu-like symptoms (*r* = 0.312, *p* = 0.064) during the last visit while on treatment. Finally, after calculating the change in scores from baseline to the last visit while on treatment, low and nonsignificant correlations were noted between the FLSI and the FSS (*r* = 0.133, *p* = 0.46) and the FLSI and the BDI-II (*r* = −0.068, *p* = 0.71).

### Known groups validity

As expected, the mean FLSI score was higher among subjects who had flu-like symptoms as reported by the investigator (mean = 4.1, SD = 6.0) compared with those whose investigator had not reported flu-like symptoms (mean = 1.4, SD = 3.9), although the difference was not statistically significant (*p* = 0.12). Mean FLSI scores were also significantly higher (*p* = 0.002) among subjects reported by their investigator to have musculoskeletal symptoms versus those with no symptoms (mean = 6.8, SD = 8.3 vs. mean = 1.0, SD = 2.3) (Table 3).

**Table 3:** Known groups validity

	Mean FLSI at baseline	*p* value
Pts with *any* investigator-reported flu-like symptoms	4.1	
Pts with *no* investigator-reported flu-like symptoms	1.4	0.120
Pts with investigator-reported musculoskeletal symptoms	6.8	
Pts without investigator-reported musculoskeletal symptoms	1.0	0.002

### Responsiveness

For the full sample, responsiveness measures represented a small effect size (SES = 0.403, SRM = 0.386), while the mean FLSI score increased from baseline to last visit among all treatment groups (Table 4).

**Table 4:** Responsiveness of the Flu-Like Symptom Index

Treatment group	*N*	Mean Flu-Like Symptom Index			SES	SRM
		Baseline	Last visit	Change		
All patients	173	3.4	5.5	+2.0	0.40	0.38
pegINF lambda	27	1.9	2.9	+1.0	0.25	0.20
pegINF alfa	36	2.8	5.6	+2.7	0.62	0.38

### Interpretation of scores

Distribution-based methods were used to compute estimates of the minimum important difference (MID) across both studies, including the SEM and 0.50 effect size. The calculated SEM was 2.61, while the 0.50 effect size was 2.52, which suggest an initial estimate of the MID for the FLSI of 2.5–3.0 points. Using a cumulative distribution function curve, we determined the cumulative proportion of the study sample that achieved a change of at least 2.5, the lower bound of the MID. Based on this, between 50 and 82 % of all subjects achieved the MID.

## Discussion

CHC infection represents a serious health problem in the USA and throughout the world. Patients with CHC typically face a treatment regimen that is commonly associated with several physical side effects including flu-like symptoms. These flu-like symptoms can impact a patient’s functioning, including productivity at work. While it is generally accepted that for many patients, some flu-like symptoms are to be expected, the severity of these symptoms is not typically measured, even with increased awareness of the importance of monitoring such side effects within CHC studies [13]. Given the fact that adverse side effects are often a primary reason for delaying, altering or even discontinuing treatment, the ability to accurately and consistently assess these symptoms’ severity can aid greatly in reducing treatment maladherence.

The HPSS-D represents the first known patient-reported outcome (PRO) measure for assessing physical symptoms experienced by patients treated for CHC and contains a scale specifically for flu-like symptoms. Our study demonstrates that the FLSI has an adequate level of validity and reliability for measuring flu-like symptoms in CHC-treated patients. We utilized multiple approaches when investigating validity and reliability, and whenever possible used data from each time point. Exploratory Factor Analysis indicates that the FLSI represents a single underlying construct. When the initial EFAs were conducted, we investigated the inclusion of an additional item regarding physical fatigue. EFA results and measurement properties for both the 4- and 5-item versions were almost identical, providing additional support that the current composition of the FLSI is appropriate. Further, the data demonstrate that the FLSI has acceptable internal consistency reliability and test–retest reliability. Convergent validity of the FLSI to several similar constructs is supported through moderate correlations between the FLSI and the investigator-reported ratings, the FSS, and by the large correlation with the BDI-II. Known groups validity of the FLSI is demonstrated based on the trend in mean scores, and although those particular results did not achieve statistical significance, the moderate sample size could have played a role. Finally, distribution-based methods estimated the MID as being 2.5–3.0.

The limitations to this study sample include the moderate size and the treatment-naïve status of all subjects. In addition, it would have been ideal to also estimate the MID using an anchor-based approach as the literature suggests that a combination of anchor and distribution-based methods is optimal, but a suitable anchor was not available.

Future research is needed to evaluate validity with more closely related constructs, assess known groups validity based on other group definitions, and to estimate the MID using anchor-based approaches. Evidence of validity among treatment-experienced patients is warranted. Also, increased statistical power would be provided by conducting these analyses with a larger sample size.

## Conclusions

Assessing the severity of flu-like symptoms in CHC patients is crucial given their association with treatment discontinuation. The HPSS-D is the only known PRO measure containing a scale assessing flu-like symptoms. This is the first examination of the measurement properties for the FLSI, and results demonstrate it has acceptable validity and reliability. The HPSS-D and, particularly the FLSI, can aid in the management of treatment for patients with CHC, potentially helping to minimize maladherence or treatment withdrawal.
